# Gait parameters are differently affected by concurrent smartphone-based activities with scaled levels of cognitive effort

**DOI:** 10.1371/journal.pone.0185825

**Published:** 2017-10-12

**Authors:** Carlotta Caramia, Ivan Bernabucci, Carmen D'Anna, Cristiano De Marchis, Maurizio Schmid

**Affiliations:** Department of Engineering, Roma Tre University, Rome, Italy; University of Western Ontario, CANADA

## Abstract

The widespread and pervasive use of smartphones for sending messages, calling, and entertainment purposes, mainly among young adults, is often accompanied by the concurrent execution of other tasks. Recent studies have analyzed how texting, reading or calling while walking–in some specific conditions–might significantly influence gait parameters. The aim of this study is to examine the effect of different smartphone activities on walking, evaluating the variations of several gait parameters. 10 young healthy students (all smartphone proficient users) were instructed to text chat (with two different levels of cognitive load), call, surf on a social network or play with a math game while walking in a real-life outdoor setting. Each of these activities is characterized by a different cognitive load. Using an inertial measurement unit on the lower trunk, spatio-temporal gait parameters, together with regularity, symmetry and smoothness parameters, were extracted and grouped for comparison among normal walking and different dual task demands. An overall significant effect of task type on the aforementioned parameters group was observed. The alterations in gait parameters vary as a function of cognitive effort. In particular, stride frequency, step length and gait speed show a decrement, while step time increases as a function of cognitive effort. Smoothness, regularity and symmetry parameters are significantly altered for specific dual task conditions, mainly along the mediolateral direction. These results may lead to a better understanding of the possible risks related to walking and concurrent smartphone use.

## Introduction

Mobile phone use is steadily increasing worldwide, with an average diffusion of 88% among adult population (43% of adults own at least a smartphone and 45% own at least a normal cellphone), and these figures are even higher among young people (ages 18–34) [1]. The presence of advanced computational and technological features, such as touch screen, built-in apps and internet access [2] within smartphones, makes it possible to help people executing many different activities [3] while moving. Thanks to these advantages, smartphones have become part of daily activities and they are often used during walking.

Walking is generally believed to be an automatic motor task [4], however it requires active cognitive areas of executive function [5] and attention [6]. Walking while using a mobile phone thus represents a dual-task and requires an appropriate division of attention [7]. It has been shown that the concurrent use of smartphone while performing other activities introduces cognitive distraction and drastically decreases field of vision and attention to the environment [8]. During walking it also reduces arm swing and alters head orientation, which may increase risk of falls [9].

Researchers have recently evaluated how different smartphones activities affect human gait. Jeon et al. [2] investigated how spatio-temporal variables of gait change in young healthy people during walking while finding applications or listening and answering questions. Demura and Uchiyama [10] observed a decrement of gait speed and stride length and an increase of stance phase when people were asked to write an email. In addition, Strubhar et al. [11] found a decrease of velocity, cadence and step length, and an increase of the double support time share during gait cycles. In Schabrun et al. [9] texting and reading while walking led to decreased speed and increased absolute lateral foot position, and texting showed higher variations in these parameters. In the study of Lamberg and Muratori [7], subjects walked straight toward a target and repeated the same path with a limited vision of the surrounding environment while talking on a cell phone and texting, with the latter showing increased lateral deviation. Along a path with obstacles, slower walking speeds, shorter step lengths, lower step frequencies and longer double support times were found when texting or math gaming [12].

These studies all confirm that smartphone use affects gait kinematics and may compromise walking stability [13,14], thus increasing the risk of falls. Indeed, the risk of falling or tripping is associated with variations of spatio-temporal parameters, such as gait speed [15], and using non-preferred gait patterns has been shown to induce an increased risk [16].

All the former studies share two common features: they were performed indoor in laboratory conditions and along short paths. Recently, Plummer et al. [17] compared the dual task effects between lab settings and real-life conditions, and found that the decrease of gait speed (the only spatio-temporal parameter analyzed) was consistent between the two scenarios. In outdoor and uncontrolled conditions, however, a quantitative assessment of the overall effect of smartphone use on a multiplicity of spatio-temporal gait features is still lacking.

The purpose of this study is thus to examine the individual effect of smartphone activities with different cognitive loads on a variety of gait parameters, including symmetry, regularity and smoothness measures. Compared to previous studies, we opted to broaden the types of activities performed with a smartphone while walking, including the most common ones, according to the recent literature [18]: interaction in a social network, playing a mathematical game, phone calling, and executing two different levels of online text chatting based on cognitive load. With this set of activities, known to display different levels of cognitive load [19], we investigated which were most perturbing to gait. By bringing the experiments outside the lab, we could simulate real-life conditions, thus minimizing possible gait alterations associated with lab settings. We were able to bypass space limitations, thus capturing the data along a distance that enabled us to extract that share of gait parameters that need longer temporal supports. In order to minimize confounding factors associated with naivety with smartphones, a population sample of young adults was monitored.

## Materials and methods

### A. Participants and procedure

Ten healthy young volunteers (age range 21–23; 4 females) with no referred motor disorders were recruited from the same university community for participating to the experiments. All of them gave written informed consent according to the declaration of Helsinki. The research study was approved by the Ethics Committee of the Applied Electronics Section of the Department of Engineering (Ref. # 01–016).

Participants were chosen based on their extended daily use of the smartphone (at least 2 hours per day) and on their dexterity in its use; all of them used their own device for the experiments. The experiment was performed in an external environment, in a pedestrian walkway in front of the lecture halls of the university where the participants usually attend courses, consisting of a 200 m long straight path to be repeatedly walked until the end of the task sequence. The path showed few irregularities on the ground and was characterized by a low pedestrian traffic, to represent a relatively quiet urban environment.

Participants were instructed as follows: they were informed on the path to follow, they were asked to use the smartphone with their preferred hand and they were told to perform a set of activities with their smartphone. Further instructions were provided during the experiment via instant message. Subjects were asked to install on their own smartphone a mathematical game and to play with it some minutes before the experiment in order to get acquainted with it. All the other required applications were already installed on all the devices, and were well known by the subjects, thus no familiarization for the other activities was needed.

The walking task sequence was kept fixed for all the subjects and included the following:

1. with no additional concurrent activities (Baseline);
2. while using an instant messaging app, answering to general knowledge questions asked by the experimenter at the other end (Low Cognitive Load—LCLtext);
3. while talking on the smartphone about daily activities (Talk);
4. while surfing on a specific page of a social network (Surf);
5. while playing a maths game on the smartphone (Math);
6. while using an instant messaging app, by reverse texting a word sent by the experimenter at the other end (High Cognitive Load—HCLtext).

In order to include at least 20 strides for a computation of a number of parameters [20], each task lasted 40 seconds, and a normal walk lasting 40 seconds was interspersed between each task to avoid short-term interference between successive tasks. To perform all these activities, each participant repeated the straight path many times. After the end of the experiment, each subject was asked if she/he experienced incidents during the experiments (stumbles, trips, staggering) and which task she/he considered as most interfering with walking. The entire experiment lasted around 20 minutes.

### B. Instrumentation and data recording

**Fig 1:**
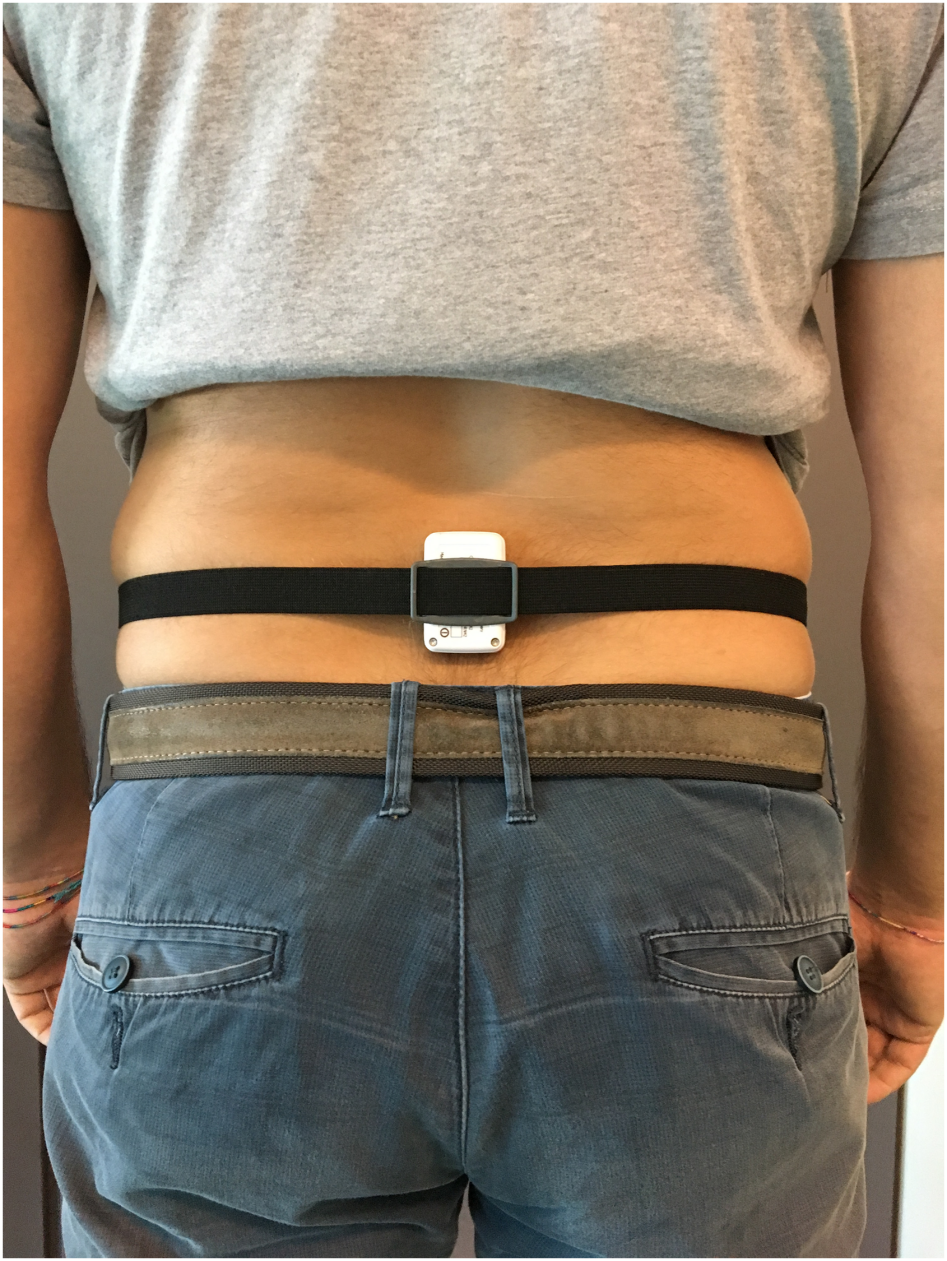
Placement of the inertial sensor on L3.

Each participant wore one wireless inertial measurement unit (Shimmer3, Shimmer sensing, Dublin, Ireland) placed on the back of the lower trunk—L3 –using an elastic belt (Fig 1) [21]. Linear accelerations along the three major axes (vertical, VT; anteroposterior, AP; mediolateral, ML) were captured at 102.4 samples/s (range ± 2 g). The data were stored for offline processing.

A voice recorder was used to store the start and end time of each task.

### C. Data processing and model for gait parameters estimation

Raw acceleration data were segmented for each task: the 20 central seconds of each task were used to extract 19 spatio-temporal gait parameters from each data segment: step time, step length, stride frequency, gait speed; then, for each component, step regularity, stride regularity, step symmetry, harmonic ratio and index of harmonicity. For Baseline, data over two 20 s long sequences were used. The methods for the extraction of spatio-temporal parameters were implemented in Matlab R2016b. The parameters were divided into four different groups, defined as follows:

- Spatio-temporal group, which included step time, gait speed, step length and stride frequency:
  ○ Step time was estimated as the half of the time lag that maximizes the data resulting from the sum of the unbiased auto-covariance of the three acceleration components in the range 0.4–4 seconds [22].
  ○ Step length and gait speed were modeled using the method proposed by Zijlstra and Hof [23] with the modification introduced by Gonzales et al. [24]. The concept of inverted pendulum is used for modelling the single stance phase, while the double stance phase is given by a value proportional to the foot length. Moreover, the estimation of initial and final contact of the foot was necessary for determining the time of occurrence of these two phases.
  ○ Stride frequency is given by the median of the modal frequencies of the spectra along the three directions [22].
- Gait regularity group, which included the stride regularity values along the three components:
  ○ Stride regularity, which indicates similarity between successive strides, is calculated from the normalized auto-correlation of the accelerometer data components over the three axes [25]. In particular, for the VT and the AP directions, it is the amplitude value of the second positive peak at lag different from zero, while, for the ML direction, it corresponds to the first positive peak at lag different from zero. Values of stride regularity closer to 1 indicate a more regular gait. A less regular gait has been linked to a less stable walking pattern [26].
- Step symmetry and regularity group, which included the step regularity, the step symmetry, and the harmonic ratio values, all calculated over the three axes:
  ○ Step symmetry describes the similarity between right and left steps; step regularity expresses the similarity between successive right (left) steps. By taking the same auto-correlation functions described before, for VT and AP components the value of the first positive peak at lag different from zero corresponds to step regularity, while the ratio between this latter value and the stride regularity expresses the step symmetry [25]. In the ML direction, the step regularity is calculated by considering the first negative peak, while the step symmetry divides the step regularity by the corresponding stride regularity defined before. For both parameters, values of VT and AP components closer to 1 –and to -1 for the ML component–indicate more symmetric and regular steps.
  ○ The harmonic ratio, another symmetry index of gait that can be calculated for the three components of acceleration, is given by the ratio between the summation of the first 10 components in phase (even) and the first 10 components out of phase (odd) of the signal for the VT and AP axes, while for the ML axis the ratio is odd to even [27]. The higher the harmonic ratio, the more symmetric the gait. In the used implementation, the harmonic ratio was considered by the decomposition of the whole signal along one task into harmonics [28], and not by considering each stride calculated individually.
- Gait smoothness group, which includes the indices of harmonicity along the three axes:
  ○ The index of harmonicity [29] can be calculated for the three components of the linear acceleration as the ratio between the power spectral density at the fundamental frequency (step frequency for AP and VT directions and stride frequency for the ML direction) and the sum of the power spectral density at the fundamental frequency and the first five super harmonics.

### Statistical analysis

Descriptive statistics were calculated for each parameter under each task condition. Multivariate Analysis of Variance was performed for each parameter group separately. Then, for each parameter, the normality of data distribution was estimated through Lilliefors test and a repeated measures univariate ANOVA test was performed to evaluate the overall effect of all the tasks on each parameter. Bonferroni correction was done and post-hoc analysis was applied to study the statistical difference between single pairs of tasks. The level of significance was set at 0.05.

## Results

Results obtained from statistical analysis show an overall significant effect of the performed tasks on the spatio-temporal parameters group (p = 8.2E-03), on the gait regularity one (p = 2.8E-4), on the step symmetry one (p < 1E-6), and also, though to a lesser extent, on the harmonicity group (p = 1.0 E-02).

By looking at each group individually, in the spatio-temporal group, step time (F = 11.01, p = 5.80E-06), step length (F = 4.92, p = 1.1E-03), gait speed (F = 10.97, p = 6.06E-07) and stride frequency (F = 9.66, p = 2.56E-06) were all significantly affected. During all dual tasks, except for Talk, step time was significantly higher than in Baseline, while the other parameters were lower.

In the gait regularity group, stride regularity along the ML direction was significantly affected by the task condition (F = 2.46, p = 4.7E-02), while the other two components were not. LCLtext was the only task showing a significant decrement compared to Baseline.

In the step symmetry group, the ML components of step symmetry (F = 3.19, p = 1.51E-02), step regularity (F = 2.83, p = 2.64E-02), and harmonic ratio (F = 3.19, p = 1.51E-02) were all significantly affected, together with step symmetry in the AP direction (F = 3.83, p = 5.7E-03). Step symmetry ML decreased during Talk and increased during HCLtext, while step regularity ML and harmonic ratio ML decreased during Talk.

For the harmonicity group, the index of harmonicity along the ML direction was significantly affected by the task (F = 6.13, p = 2.1E-04); more specifically, it increased during LCLtext and HCLtext.

Apart from step symmetry in the AP direction, no significant differences were observed in the sagittal plane (i.e. along the VT and AP directions).

**Fig 2:**
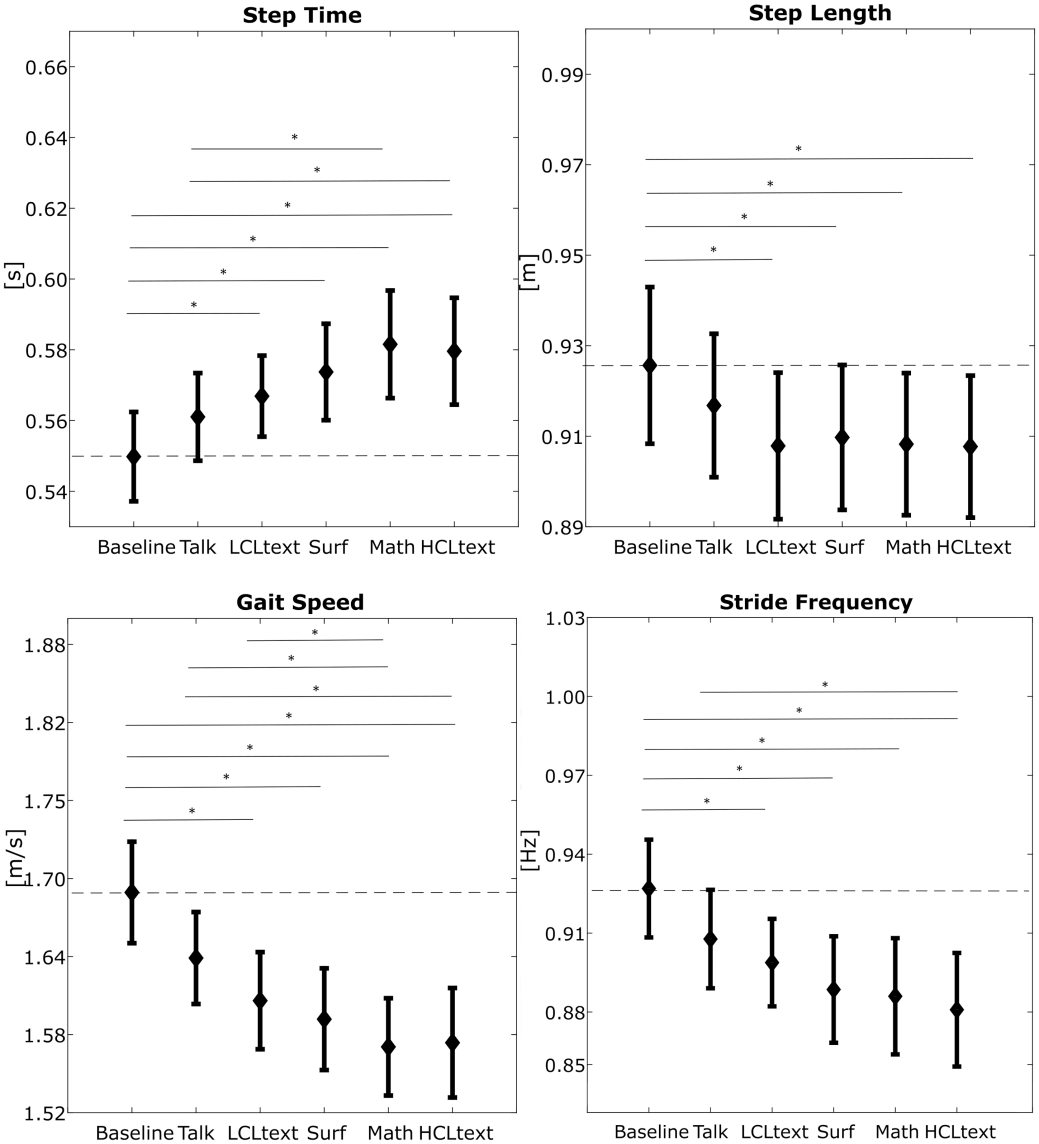
Means ± standard errors of the spatio-temporal parameters that showed significant differences among tasks. The straight lines with * indicate the significant difference between tasks.

**Fig 3:**
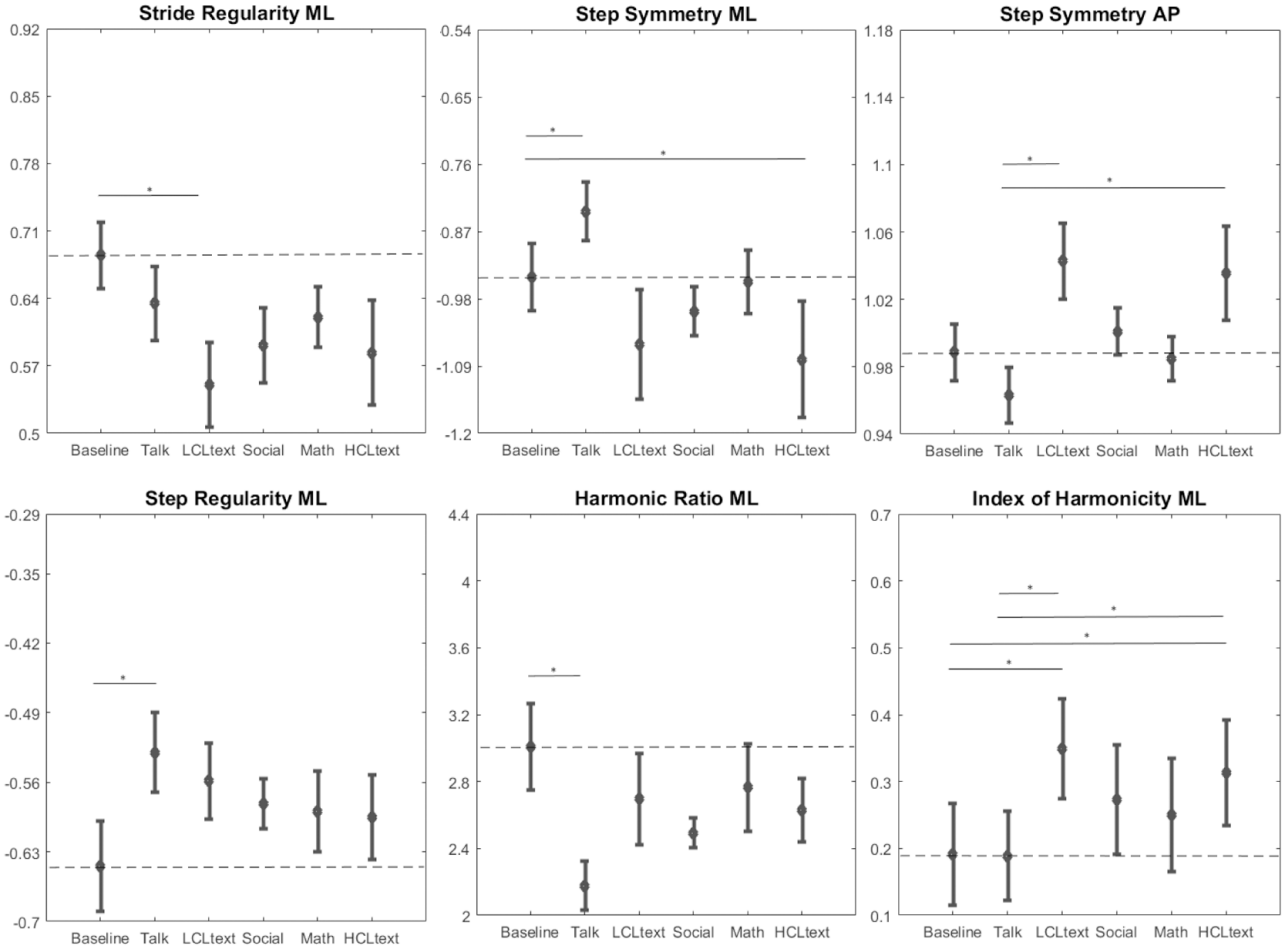
Means ± standard errors of the gait regularity, symmetry and smoothness parameters that showed significant differences among tasks. The straight lines with * indicate the significant difference between tasks.

In Figs 2 and 3, means and standard errors of each parameter under each task are shown. The pairwise statistical differences between tasks are indicated by straight lines with stars.

Concerning the differences between tasks excluding the Baseline condition, we found that HCLtext induces significant differences as compared to Talk in the following parameters: step time, gait speed, stride frequency, step symmetry and index of harmonicity in the ML direction, and step symmetry in the AP direction. Also, Math determines a significant increase of the step time and a concurrent decrease of gait speed as compared to Talk. LCLtext shows a significant increase of the index of harmonicity in the ML direction as compared to Talk.

As shown in literature [11], we found that texting, which is present in both LCLtext and HCLtext, leads to a significant decrease of stride frequency, step length and gait speed, and an increase of step time. In this study, we consider texting as an instantaneous and continuous typing needed to answer messages sent from the experimenter, as happens in real life during a chat. We found the same results in Math and in Surf, while Talk did not show significant differences in any of these parameters. The parameter values obtained in Talk conditions are not significantly different from Baseline, except for step regularity and harmonic ratio in the ML direction.

## Discussion

The presence of an additional concurrent cognitive task is agreed in the literature to produce alterations of gait. This has been demonstrated a number of times both in lab-based conditions, and in some cases also in outdoor environments. The results obtained in this research study confirm these observations and utilize longer distances. Restricting the analysis to tasks that involved smartphones and analyzing several gait parameters, we found that smartphone use makes people walk slower, due to a combination of lower stride frequency and shorter steps. Moreover, concurrent smartphone use makes gait less regular, with a lower level of symmetry. For some tasks, it also showed higher harmonicity than in the normal walking.

Some of our results regarding frequency, lengths, and durations are in accordance with the literature and have been explained as the result of a higher effort required in the execution of both tasks. While being historically seen as an automatic task, the involvement of cognitive function in gait is being increasingly acknowledged in the literature [30], and the results obtained in the present research study conducted on young adults, proficient with smartphone use, confirm this hypothesis. An interesting addition to these considerations can be linked with the evidence that, for most spatio-temporal parameters (step time, step length, gait speed, stride frequency), there is a uniform trend function of the cognitive effort associated with the concurrent task. Even if we cannot exclude that the absence of randomization in the task sequence may play a role (even though the blocks of normal walking were interspersed between consecutive dual tasks), this may indicate that not only a cognitive task affects gait strategies, but that its effect on the characteristics of gait is proportional to the amount of cognitive load associated with it. Specifically, the ascending order of cognitive load is the following: phone talking, low cognitive load text chatting, surfing on social network, playing math game, high cognitive load text chatting (where the last two seemed as introducing similar cognitive efforts). Furthermore, at the end of the experiment subjects were asked which was the most challenging task performed: all the subjects except two identified high cognitive load text chatting as the most challenging. Our results confirm these reports, as this activity was most often significantly different from the baseline and from phone talking. Regarding harmonicity of gait, we did not observe a correspondence in proportion to cognitive effort: gait appeared more harmonic when cognitive load is present in text chatting activities, and not for the other dual task ones, while this did not significantly appear for surfing and math gaming. All these activities, in fact, require to hold the smartphone in front of the subject at the trunk height, and to focus on the screen. We hypothesized that this would lead to a more harmonic gait pattern, as the result of minimizing relative displacements between the head-trunk complex and the smartphone. However, the two text chatting activities require typing continuously on the smartphone as compared to the more interspersed typing when web surfing or math gaming. We speculate here that this difference in “duty cycle” might lead to have only the two text chatting activities significantly different from walking alone. Moreover, talking while walking does not remarkably limit frontal vision, and this may also justify why harmonicity in the ML direction was not significantly different from walking alone.

Phone talking while walking did not lead to significant differences in spatio-temporal parameters either: among the monitored activities, it is the most common one executed while walking and it does not introduce notable limitations to the vision of the environment. However, it leads the head to bend in one direction–and, as a consequence, also the trunk, slightly–to fit its inclination. Moreover, arm swing is altered [31], as one arm natural swing is restricted by keeping the phone close to the ear; overall, the center of mass moves to the side where the phone is held, thus leading to a higher gait asymmetry, causing a lower harmonic ratio along the mediolateral direction [32–34], as the consequence of the increased presence of odd harmonics relative to the even ones [35]. This may be why step regularity and symmetry along the ML direction appeared different when talking as compared to baseline. It has been suggested that, despite the name, that harmonic ratio is a measure of step-to-step symmetry (and, indirectly, regularity), more than an indicator of harmonicity or smoothness [32].

Texting is a secondary task which instead combines the use of higher amounts of visual [36], motor and cognitive resources, with a higher level of difficulty than a phone conversation performed while walking [9,12,37]. Furthermore, a recent study showed that the change in walking, during texting, may be attributed to the altered visual search behaviour [8]: the subjects may not be able to acquire sufficient visual information using the central visual field to plan direction of the walk with an increase of the risk of falling.

Another interesting consideration comes from the evidence that we observed significant effects in the mediolateral direction: the attention resources to ML direction control of the trunk are agreed in the literature to be higher than those required for the control of the other components [38,39], which may be passively regulated with minimal attentional control. It is thus no surprise that the cognitive effort had more of an effect on parameters captured along this specific component. The instability of mediolateral control, that can be reflected into the variability of some of the corresponding gait parameters, is associated with an increased risk of falling [40]. Regarding the index of harmonicity, the obtained results confirm the observed negative correlation with gait speed, i.e. slower walking is smoother [29]. On top of this, in the ML direction smoothness is not an indicator of stability: in fact, elderly people who display higher harmonicity in the ML direction are at increased risk of falling [41]. If this evidence applies also to younger people, we could interpret the higher values of index of harmonicity displayed when texting as a marker of gait instability.

Moreover, it is worth noting that the vast majority of parameters in the sagittal plane did not show significant differences when performing a dual task. We speculate that, in presence of a dual task, subjects tend to adjust the spatial and temporal components of gait (i.e. speed, stride frequency, step length and step time), in order to preserve a regular, symmetric and smooth gait along the sagittal plane.

Based on the previous considerations, the analyzed dual tasks seem to induce a considerable deviation from the preferred gait patterns adopted during natural walking (i.e. the adopted combination of spatio-temporal gait parameters), and this might have implications for the related risks. For instance, walking with a non-preferred and externally-imposed gait cadence has been shown to be highly correlated to increased metabolic cost and decreased stability [16], reduced reaction time [42], and unwanted additional veering when walking along a straight path with a reduced field of vision [43]. We hypothesize that such variations could be also present, and with varying extent, when stride frequency is altered as a consequence of dual task execution with different levels of cognitive engagement.

The simultaneous performance of two tasks implies a competition for attention resources and requires that the brain unconsciously decides which task to prioritize when no instructions are given about task prioritization [30]. During our experiments, no instructions were given to subjects so that, during dual task demands, they were free to choose their ‘default’ prioritization scheme [30,44]. In this way, we could study how they behave during dual task conditions, without other external interferences. Some studies [17,30] explored how prioritization affects gait in young and older people: it was found that task prioritization alters gait speed more in young adults than in elderly, suggesting a reduced ability to prioritize cognitive tasks caused by a decline in mental flexibility due to age [30].

The reduced sample size could limit the power of the analysis and prevented us from dividing the sample group based on risk propensity: in the future, it would be interesting to link differences in young cohorts between risk-takers and risk-avoiders, to check whether these two classes display different gait behaviour when dual tasks are present. Another future direction involves the study of such gait alterations in a population which is both non-proficient in smartphone use and potentially affected by a reduced mental flexibility and physical decline, such as the elderly. These characteristics interfere with gait performance [45], resulting in higher probability of falling [46].

## Conclusions

This study extends the number of monitored activities performed with smartphones, including the most common ones in young people's everyday life, such as surfing on a social network, text chatting, playing a game and phone talking [3]. We had the possibility to characterize the effect of each of these activities on most gait parameters, recorded along a long-duration experiment and in real life conditions. Knowledge of how walking parameters vary due to smartphone use may allow us to understand the possible associated risks.

We are able to confirm that, even for young adults who are proficient with smartphone use, gait behaviour is affected in a significant way for those activities that require a mid-to-high cognitive effort. These modifications are not limited to the spatio-temporal characteristics of gait, such as reduced step length or gait speed, or to mechanical alterations directly associated with the smartphone use, but impact regularity, symmetry and smoothness of gait as well (mostly along the mediolateral direction).

Further studies, possibly including people with different cognitive reserve, may help shed light on the direct and indirect risks associated with these adjustments.
