# Correction: Gait parameters are differently affected by concurrent smartphone-based activities with scaled levels of cognitive effort

**DOI:** 10.1371/journal.pone.0193258

**Published:** 2018-02-15

**Authors:** Carlotta Caramia, Ivan Bernabucci, Carmen D'Anna, Cristiano De Marchis, Maurizio Schmid

Fig 2 is incorrect. The authors have provided a corrected version here.

**Fig 2:**
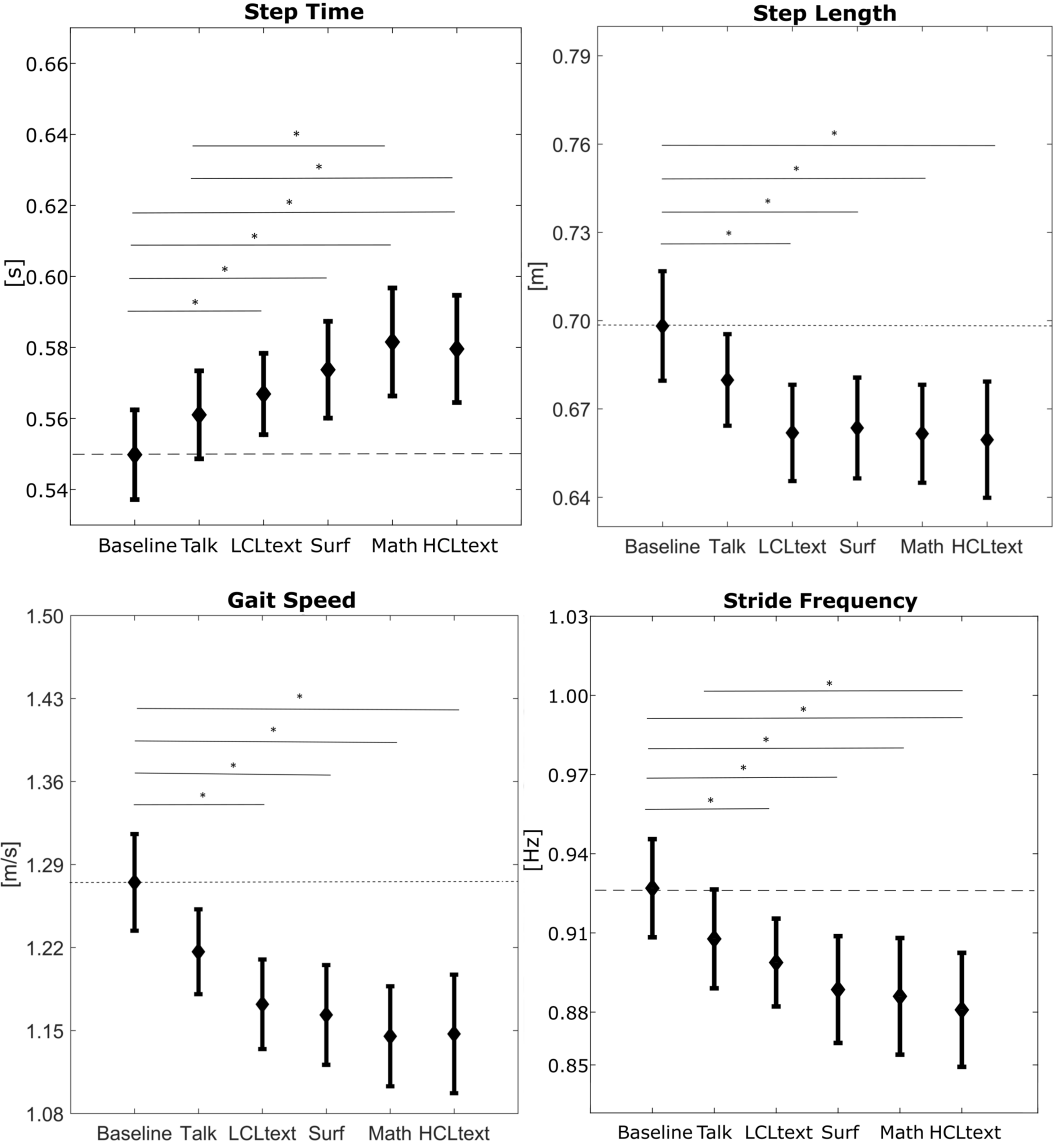
Means ± standard errors of the spatio-temporal parameters that showed significant differences among tasks. The straight lines with * indicate the significant difference between tasks.
